# Alternative RNA splicing: contribution to pain and potential therapeutic strategy

**DOI:** 10.1016/j.drudis.2016.06.017

**Published:** 2016-11

**Authors:** Lucy F. Donaldson, Nicholas Beazley-Long

**Affiliations:** School of Life Sciences and Arthritis Research UK Pain Centre, University of Nottingham, Nottingham NG7 2UH, UK

## Abstract

Since the sequencing of metazoan genomes began, it has become clear that the number of expressed proteins far exceeds the number of genes. It is now estimated that more than 98% of human genes give rise to multiple proteins through alternative pre-mRNA splicing. In this review, we highlight the known alternative splice variants of many channels, receptors, and growth factors that are important in nociception and pain. Recently, pharmacological control of alternative splicing has been proposed as potential therapy in cancer, wet age-related macular degeneration, retroviral infections, and pain. Thus, we also consider the effects that known splice variants of molecules key to nociception/pain have on nociceptive processing and/or analgesic action, and the potential for control of alternative pre-mRNA splicing as a novel analgesic strategy.

## Highlights

• Alternative pre-mRNA splicing generates multiple proteins from a single gene.
• Control of alternative splicing is a likely therapy in cancer and other disorders.
• Key molecules in pain pathways – GPCRs and channels – are alternatively spliced.
• It is proposed that alternative splicing may be a therapeutic target in pain.

## Teaser

The control of alternative splicing is a growing area of investigation for potential therapy. Here we consider what is known about alternative splicing and its control in relation to pain.

## Introduction

The first description of alternative splicing relating specifically to nociceptive systems was the production of calcitonin gene-related peptide (CGRP) encoded by the calcitonin gene [1], a neuropeptide intimately associated with nociception and inflammation. Since then, multiple molecules have been identified in which alternative splice variants might contribute to the regulation or modulation of nociception, and a limited number have received a great deal of attention. Unfortunately, despite identification of alternatively spliced isoforms of key molecules in nociception, such as ion channels, G-protein-coupled receptors (GPCR), or growth factors, how splicing affects function in the nociceptive system is largely unknown. Inadequate tools to investigate functional changes, for example antibodies and pharmacological agents that do not distinguish between splice isoforms, hinder these investigations. Despite the technical hindrances, it is known that alternative splicing of, for example, GPCR pre-mRNA, can alter receptor pharmacology by affecting ligand specificity and potency [2], receptor trafficking and internalisation [3], and regional and cellular expression 4, 5. Ion channels are fundamental to the function of nociceptive neurons. They regulate neuronal excitability, neurotransmitter release, and control sensory transduction at peripheral nociceptive terminals [6]. Many ion channel families comprise multimers of different subunits, including pore-forming units, and, in some cases, accessory units. Therefore, there is enormous scope for alternative splicing to modulate channel function, and pharmacology, and thereby affect pain.

**Figure 1:**
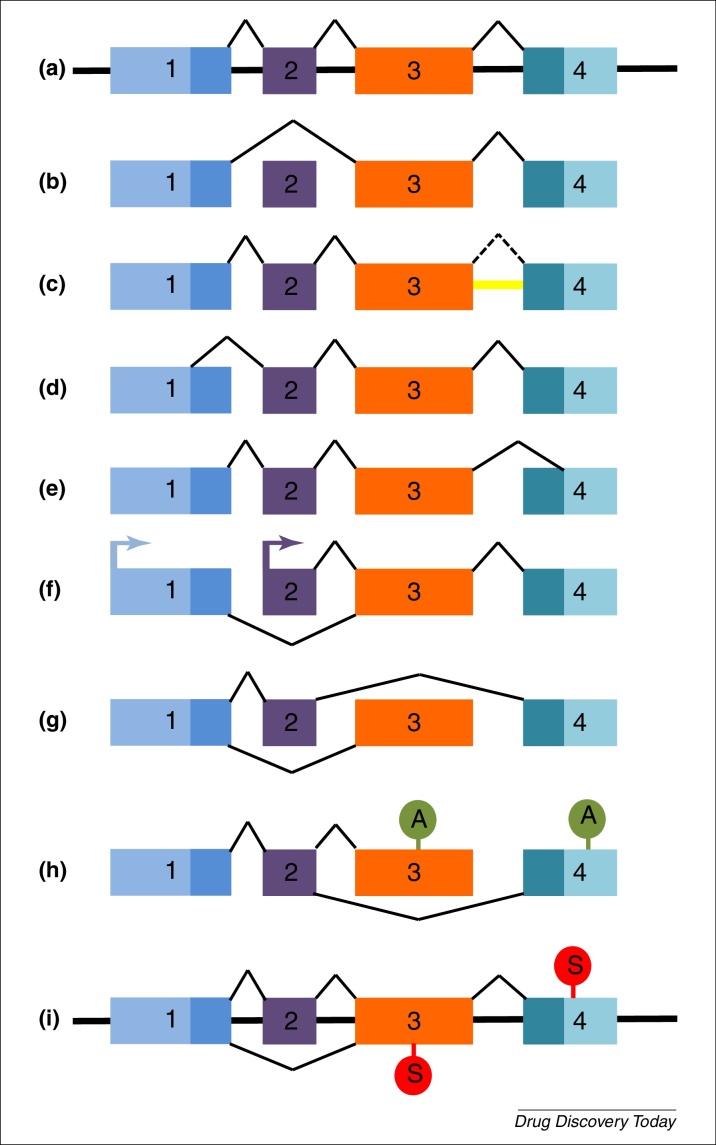
Different types of alternative splicing compared with constitutive splicing. **(a)** Genomic structure showing the constitutive splicing of a pre-mRNA containing four exons, coloured boxes denote different exons. Alternative splice variants can arise by different mechanisms, such as: **(b)** exon skipping. Exon 2 is spliced out of the pre-mRNA; **(c)** intron retention. The intron between exons 3 and 4 (yellow) is not spliced out (dotted line) and is included in the mature mRNA; **(d)** alternative 5′ donor site. An intra-exonic splice site in exon 1 is used and gives an alternative 5′ sequence; **(e)** alternative 3′ acceptor site. An intra-exonic splice site is used in exon 4 and gives an alternative 3′ sequence; **(f)** alternative promoters; **(g)** mutually exclusive exons, where two mature mRNAs exist containing either exon 2 or exon 3 but never both exons together; **(h)** alternative polyadenylation sites; or **(i)** alternative stop codon use. Top stop codon: Constitutive splicing leads to use of stop codon in exon 4. Bottom stop codon: Exclusion of exon 2 leads to alternative stop codon in exon 3 and nonsense-mediated decay.

Alternative pre-mRNA splicing (a.k.a. alternative splicing) is the mechanism through which intronic RNA is removed from the pre-mRNA and the exons are joined in the mature mRNA. It differs from constitutive splicing in that alternative exons might or might not be included, and there may be exon skipping, intron retention, alternative 5′ and 3′ splice sites and mutually exclusive exons [7] (Fig. 1), leading to the generation of potentially hundreds of proteins from a single mRNA. In some instances, multiple mRNAs are generated that all lead to the translation of the same protein product, for example brain-derived neurotrophic factor (BDNF) [8]. Functionally, expression of alternative splice variants can contribute tissue-specific expression patterns (e.g., BDNF), can alter neuronal mechanisms, such as neurotransmitter release (e.g., voltage-gated calcium channels; VGCC), or modulate cellular survival or function (e.g., neurotrophic factors). Relatively subtle changes in protein structure brought about through alternative splicing can dramatically alter function; for example, pro- and antiangiogenic forms of vascular endothelial growth factor that differ in only six amino acids.

Alternative splicing is only one mechanism through which gene expression is controlled, but it is an important one. This process must be extremely precise, because splicing at sites even one nucleotide out of place can result in shifts in the open reading frame, production of nonfunctional or aberrant proteins, or unstable transcripts through the introduction of premature stop codons leading to nonsense-mediated decay [9]. Given the required precision of alternative splicing, it is perhaps unsurprising that it is estimated that up to 50% of disease-causing mutations in the human genome affect splicing 9, 10. To achieve precision, the spliceosome, a complex of approximately 170 RNA-binding proteins and small nuclear RNAs in complex forming small nuclear ribonucleoproteins (snRNPs), must accurately recognise intron and/or exon boundaries through recognition of specific splicing sequences [10].

The precise understanding of the alternative splicing mechanisms of specific genes is fairly limited. General mechanisms that are understood include the presence of other splicing regulatory proteins, such as the serine-arginine-rich (SR) family of kinases, and heterogeneous nucleoribonuclear proteins [11]. Tissue-specific expression of splicing factors, such as Nova1 and 2, or Fox proteins, adds further complexity to the control of alternative splicing and RNA maturation. Nova proteins are, for example, particularly important in the regulation of RNAs contributing to synaptic function [12].

In this review, we consider what is known about alternatively spliced variants of GPCRs (particularly mu opioid receptors), ion channels (particularly VGCCs), and growth factors, for which evidence supports or suggests a contribution to nociceptive processing. We also speculate briefly on the effects that known splice variants of key molecules might have on nociception where no investigations have yet been published. Finally, we consider whether the control of alternative splicing is a potential analgesic target.

## GPCRs, alternative splice variants, and pain

Bioinformatic studies indicate that approximately 50% of GPCRs are intronless and the remainder can undergo alternative splicing [13]. For most identified alternatively spliced isoforms, it is not known how the change of mRNA and, therefore, protein sequence affects receptor function, because most information derives from RT-PCR mRNA rather than from functional studies. The question of how alternative splicing of nociception-associated GPCRs affects pain and analgesia remains.

### Mu (μ) opioid receptors

Opioid receptor agonists, such as morphine, mimic the action of endogenous opioids (encephalin, dynorphins, and beta-endorphins) and suppress neuronal excitability by affecting ion channel function, for example through the inhibition of VGCCs [14], or activation of G-protein-coupled inwardly rectifying potassium channels [15]. Although opiates are widely used analgesics, not all patients in need of pain relief respond in the same manner to these drugs. Patients can be insensitive, develop tolerance, or suffer adverse effects, such as nausea and vomiting, to one opiate yet respond effectively to another. Response to opiates has a clear genetic component because, for example, CXBK mice are insensitive to morphine yet respond to fentanyl, heroin, methadone, and morphine-6β-glucuronide [16].

Of the three opioid receptor subtypes mu (μ), delta (δ) and kappa (κ), μ undergoes extensive alternative splicing. The human *OPRM1* gene encoding the μ receptor has 12 exons with expression driven by two independent promoters. Alternative splicing varies both the number of transmembrane (TM) domains (with 1, 6, or 7 TM domains), and the length and sequence of the N and C termini, and includes exon inclusion and/or skipping, alternative 3′ splice selection, and intron retention (Fig. 1) [17].

**Figure 2:**
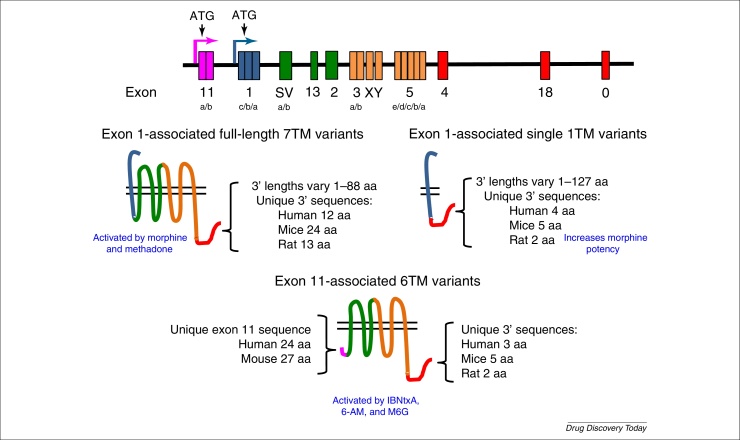
Alternative splicing of the human mu opioid receptor, MOR-1. MOR-1 is extensively spliced in different ways in rat, mouse, and human, leading to multiple receptors, including isoforms with seven, six, and one transmembrane (TM) domains. The figure shows the genomic structure (not to scale) with alternative start sites and exon structure, and examples of different splice variants protein structures. Colours in the lower panels correspond to the exons from which each part of the protein is transcribed and/or translated. The numbers (e.g., human 12 aa) denote the length of the unique C terminus in splice variants from specific species in amino acids. Note that not all known splice variants are shown here [19]. For full coverage of the multiple splice variants of MOR-1 and the species differences, see multiple publications by G.W. Pasternak.

*Source*: Adapted from [123].

The most obvious way in which μ receptor alternative splicing might regulate function is in response to opiate analgesics, most of which are targeted to the μ receptors, and this is indeed the case [17]. Exon-targeted disruption within *OPRM1* has identified regions critical for analgesia produced by different opiates; for example, morphine requires the inclusion of exons 1, 2, and 3. Isoforms excluding exon 1 are not inhibited by morphine, but are activated by the heroin metabolite, 6-acetylmorphine and morphine-6β-glucuronide, although with a reduced potency [18]. Novel molecules that target the 6TM domain truncated variant MOR have been described as potent analgesics, with no respiratory depression, dependence, or rewarding effects (Fig. 2), and analgesia can be rescued in μ receptor-knockout (KO) mice with these truncated forms [19]. Alternative splice variants are also associated with differences in opioid dependence [20] and morphine tolerance [21]. Thus, understanding μ receptor alternative splicing has already been shown to be important in understanding opiate pharmacology and in the development of new analgesics.

In other areas, the effects of μ receptor splicing are not yet clear. Alternative splicing of the μ opioid receptor can affect regional and cellular expression, and the engagement of intracellular signaling pathways. μ opioid receptor splice variants are regionally distributed in the central nervous system (CNS), with mMOR-1C being predominant in the thalamus, mMOR-1D abundant in the cortex, brain stem, and periaqueductal grey, and mMOR-1E predominant in the striatum and hypothalamus [5]. Given the different pharmacology, such regional distributions could underlie observed differences in adverse effects experienced, such as nausea and vomiting.

In the superficial laminae of the dorsal horn, μ variants containing exons 7, 8, or 9, and, hence, different intracellular C termini, are localised to presynaptic terminals expressing CGRP, whereas exon 4-containing variants are expressed equally across pre- and postsynaptic neurons, but not in CGRP-expressing neurons [22]. Alternative splicing varies the length of the μ opioid mRNA 3′ untranslated region (UTR) and the translated 3′ tail from that of two amino acids in mMOR-1B5 to 88 amino acids in mMOR-1U. The C termini of μ receptors contain the protein kinase sites *β*-adrenergic receptor kinase, protein kinase C, cAMP- and cGMP-dependent protein kinase, and casein kinase II [17]. Thus, alternative splicing in the μ opioid 3′ UTR is likely to affect the processing, localisation, and interactions of both the message RNA and the translated protein, in turn regulating receptor function.

### Metabotropic glutamate receptors

All metabotropic glutamate receptors (mGluR1–8) except mGluR2 are reported in the literature to have expressed splice variants, although genome database interrogation suggests that mGluR2 also has alternative spliced transcripts. Differential splice variant expression of group I mGluRs (mGluR1 and 5) has been identified in the dorsal horn [23]. The human gene encoding mGluR1 has at least four C-terminal splice variants with varying pharmacological properties and a dominant-negative truncated isoform containing the extracellular ligand-binding domain without the canonical 7TMDs [24]. Other truncated mGluRs, also predicted to lack the 7TMDs, have been reported and some of these are postulated to act as secreted soluble receptors or dominant-negative isoforms.

A knock-in mutant of the mGluR7 splice variant mGluR7a that lacks the PDZ domain showed impaired PKC-dependent autoinhibition of glutamate release, spatial working memory deficits, and increased susceptibility to pentylenetetrazole, but no effect on pain behaviour or anxiety was observed, indicating that the correct function of this isoform is not required for normal nociceptive processing [25]. Besides this study, little is known about how differential isoform and consequent domain expression of mGluRs in the CNS affects pain processing and pain states. Promoting the generation of dominant-negative mGluR isoforms over fully functional receptors or vice versa, depending on the mGluR subtype in question, could be an alternative analgesic strategy to receptor activation and/or antagonism or allosteric modulation.

### Cannabinoid receptors

Two cannabinoid receptors have been identified to date, CB1 and CB2, and nonselective CB receptor agonists can reduce pain sensitivity in humans and animal models [26]. There are three CB1 alternative splice variants with varying N terminal sequences. Initial investigations suggest that CB1 splice variants have different pharmacological properties in response to endocannabinoids or synthetic ligands [27], but how these differences might impact pain relief and the unwanted psychoactive adverse effects from cannabinoids is unknown.

In normal physiology, CB2 is expressed at a lower level than CB1 in the CNS. In experimental models of neuropathic and inflammatory pain, CB2 expression is induced in spinal microglia, perivascular cells, and C-fibre primary afferents [28]. CB2 agonists can inhibit associated pain behaviours [29]; thus, CB2 is an attractive target for the treatment of chronic pain. The human CB2 gene has two reported splice variants generated through tissue-specific promoter use. CB2A mRNA is detected at a higher level in brain than is CB2B, which is predominantly expressed in spleen and peripheral tissues [4]. Controlling the splicing could enhance CB2 expression in desired tissues and/or cells, while suppressing expression in cells and/or tissues where CB2 activation might have adverse effects, for example to favour primary afferent or dorsal horn spinal neuron expression in peripheral neuropathic pain states.

### Serotonin receptors

Fifteen genes encode seven serotonin subfamilies (5HT1–5HT7) classed on pharmacological properties, amino acid sequences, gene organisation, and second messenger coupling pathways. Six of the subfamilies are GPCRs and splice variants are reported for all families [30]. Serotonergic pathways are important in the descending modulation, both inhibitory and facilitatory, of spinal nociceptive processing [31].

The serotonin receptor 2C (5HTR2C) splice variants have received more attention than other 5HT receptors because, in addition to exon 5b skipping producing mRNA species containing exon 5a with or without 5b and an inactive truncated form of 5HTR2C [32], 5HTR2C is subject to further RNA editing in the form of base conversions (such as adenosine to inosine, and others). These conversions also have an effect on the splicing events. Fully unedited mRNA generates a constitutively active 5HTR2C isoform that can function without the presence of serotonin, whereas inefficient RNA editing leads to increased exon 5b skipping, thereby promoting the generation of the inactive form. 5HTR2C contributes to endogenous analgesia in the dorsal horn in neuropathic pain states, and splicing and/or RNA editing of this receptor under these conditions seems to favour those splice variants with the highest agonist-independent and agonist-induced activity [33].

### Gamma-aminobutyric acid type B receptors

Gamma-aminobutyric acid type B (GABA_B_) receptor splice variants show differential expression patterns in the CNS [34] GABA_B_ receptor agonists, such as baclofen, are used to treat alcohol dependency, muscle spasms and spasticity, and neuropathic pain. Chronic alcohol abuse leads to aberrant splicing of the GABA_B_ ligand-binding subunit (GABA_B1_) in the prefrontal cortex [35], which is postulated to reduce GABA_B_ receptor function in response to ligand, increasing the dose of agonist required in the clinic for effective treatment. It is unknown whether the splicing of GABA_B_ receptor is altered in other painful neuropathies.

### Adrenergic receptors

Alpha-adrenergic receptor (α-AR) agonists, such as clonidine, are well known for having analgesic and anaesthetic qualities, and beta-adrenergic receptors (β-AR) are also promising therapeutic targets for pain management because β2-AR isoforms are expressed by primary afferent neurons within the dorsal horn of the spinal cord and are critical for the antinociceptive actions of antidepressant drugs [36]. However, of all the adrenergic receptors, only the genes encoding α1-AR and β3-AR 37, 38 contain introns enabling alternative splicing, which in the case of β3-AR affects G-protein coupling. Nevertheless, the question of whether alternative splicing affects the antinociceptive properties of adrenergic receptor pharmacology remains unexplored.

### Prostaglandin E_2_ EP3 receptors

Prostaglandin E_2_ (PGE_2_) is synthesised by cyclooxygenase and can facilitate pain processing through four prostaglandin E2 receptors (EP1–4), of which only the EP3 subtype has alternative splice variants [39]. In the periphery, PGE_2_ affects neuronal excitability through EP1, EP3, and EP4 receptors and also modulates central nociceptive processing. Alternative EP3 splice variants differ in their C-terminal sequences, which affects both downstream signaling through either excitatory or inhibitory G proteins, and receptor internalisation [40]. For example, EP3α activation, an inhibitory G protein-coupled splice variant, can reduce both mechanical stimulus sensitivity and PGE_2_-induced spinal neuron excitability [41]. Unfortunately, little is known about the different contributions of EP3 receptor splice variants (as opposed to the EP subtypes) to nociception and how they might be exploited to, for example, reduce the adverse effects associated with current clinical cyclooxygenase inhibition.

## Ion channels, alternative splice variants, and pain

### VGCCs

The contributions of VGCC to nociception and other functions have been comprehensively reviewed many times (e.g., [42]); thus, we provide here overview of selected splice variants of Ca_v_ channel alpha subunits that have been particularly associated with nociception/pain. Ten Ca_v_ genes encode the pore-forming alpha subunits of the Ca_v_ channels, with potentially thousands of splice variants giving rise to a huge number of calcium channels. The different alpha subunit splice variants confer different properties in the channels, through different tissue localisations and/or channel properties [43].

High VGCCs require strong depolarisation of cells to be activated. Of the many different Ca_v_ types, the high voltage L, N, P/Q, and R types have been implicated in the neuronal processing of nociception. The majority of these Ca_v_ types are known to have splice variants, particularly the N and R types that confer different properties on nociceptors [44]. The alpha subunits Ca_v_2.1 [P/Q (Purkinje/granule cell)-type], Ca_v_2.2 [N (neuronal) type] and Ca_v_2.3 [R (toxin-resistant) type] are all expressed in sensory neurons and contribute to the presynaptic control of neurotransmitter release from nociceptive primary afferents, and the postsynaptic activation of spinal neurons.

### Ca_v_2.2 (N-type) VGCC

The N-type calcium channel Ca_v_2.2 is expressed on the central [45] and peripheral [46] terminals of the peptidergic group of nociceptive primary afferents and controls the release of the excitatory neurotransmitter and/or modulators glutamate and substance P. The splice variants of N-type calcium channels have been studied in great depth over several years, particularly with respect to the alternatively spliced exons (E)37a and b, and the effects that the inclusion of E37a or E37b have on channel properties and cellular events.

N-type calcium channels are subject to inhibition by GPCR-dependent pathways, for example, activation of presynaptic μ opioid receptors inhibits the opening of Ca_v_2.2, thereby reducing the release of neurotransmitter [47]. The alternative splicing of the mutually exclusive exons 37a or 37b leads to two characterised Ca_v_2.2 isoforms that differ by 32 amino acids at the C terminus. The E37a variant is enriched in nociceptive afferents, has higher expression levels on the cellular membrane, increases current density, and has longer opening times [48]. Ca_v_2.2E37a is also subject to stronger inhibition by activation of presynaptic opioid [49] and GABA receptors compared with the E37b isoform, which is voltage independent. Thus, this alternative splicing of Ca_v_2.2 renders nociceptive afferents more sensitive to modulation by GPCRs and, hence, controls the sensitivity of neurotransmitter release from these neurons at their first spinal synapse. This is important for pain because non-isoform-selective pharmacological inhibitors of Ca_v_2.2 (e.g., w-conotoxin MVIIA; a.k.a. ziconotide) can block both inflammatory and neuropathic pain in animal models [50]. However, nerve injury and inflammation variably reduce 51, 52 or increase [53] the expression and/or function of Ca_v_2.2 channels, contributing to the generation of chronic pain in addition to affecting the actions of spinally acting analgesics that modulate Ca_v_2.2 subunits [54]. The generation of isoform-specific pharmacological tools could lead to more effective pain control through direct Ca_v_2.2 blockade or possibly through the potentiation of opioid analgesia.

### Calcium channel beta and alpha2delta subunits

The α2δ calcium channel subunits have generated a large amount of interest as the target of gabapentin, an analgesic used in the treatment of neuropathic pain. At least two of the α2δ subunit family, α2δ-1 and 2, have multiple splice variants [55]. α2δ subunits are thought to contribute to the trafficking and function of Ca_v_2.2 under nerve injury conditions, and are necessary for the development of neuropathic pain [56]. There are five reported splice variants of α2δ-1βin sensory neurons, two of which, ΔA + B + C and ΔA + BΔC, are upregulated in injured neurons following traumatic injury [57]. These both form functional calcium channels with similar properties, but the shorter form has lower binding affinity for gabapentin. The authors speculate that the variable patient response to gabapentin might be related to altered expression levels of this particular splice variant. Gabapentin can also exert its effects on calcium channel trafficking through binding of a splice variant of the beta4 subunit [58]. There are four beta subunit genes, each of which also has splice variants [59]. The function and possible relevance to nociception of other beta subunit splice variants are not currently known.

### R-type (Ca_v_2.3), L-type (Ca_v_1.1–1.4), and P/Q-type (Ca_v_2.1) VGCC

There are six known splice variant isoforms of Ca_v_2.3 [60] (Ca_v_2.3a–f). Ca_v_2.3e is the major isoform expressed in spinal and trigeminal sensory neurons, being found largely in the nociceptive TrkA-expressing subgroup [60], where it also contributes to the control of spinal neurotransmitter release [61]. Pharmacological blockade of Ca_v_2.3 has implicated these channels in spinal nociceptive processing, particularly in neuropathic pain [51] and opiate analgesia [62]. Ca_v_1 and Ca_v_2 channels are important in the regulation of membrane excitability, and are extensively spliced, but there are no studies that show any clear association between L-type (Ca_v_1.1, 1.2, 1.3, and 1.4) or P/Q-type (Ca_v_2.1) calcium channels and nociception, other than an association of Ca_v_1 with familial hemiplegic migraine [63].

The large family of proteins that together comprise calcium channels is notable for having many splice variants. Although some of these splice variants have been characterised, we are still a long way from a full understanding of how pain states might affect calcium channel splicing, or how different calcium channel subunit splice variants might affect nociceptive processing. However, these examples show how understanding splice variant function can further the development of pharmacological approaches to pain control.

### Voltage-gated sodium channels

Voltage-gated sodium channels (VGSC) are major determinants of neuronal excitability, determining the depolarisation phase (up-stroke) of the action potential. Similar to VGCC, VGSC comprise different subunits: the pore-forming alpha subunit, and one or more beta subunits. Mutations in certain VGSC are now known to cause pain syndromes, congenital insensitivity to pain, or neuropathies [6]. The VGSC are usually considered in two groups, the tetrodotoxin (TTX) sensitive (Na_v_1.1–1.4, Na_v_1.6, and Na_v_1.7) and the TTX resistant (Na_v_1.5, Na_v_1.8, and Na_v_1.9). Alternative splice variants of both TTX-sensitive and -resistant groups have been reported, but the functional consequences have been described for only a few of these splice variants 64, 65.

Nerve injury results in the downregulation of most VGSC, except for Na_v_1.3, which is upregulated 64, 65. The upregulation appears to be limited to the originally described transcript rather than to a longer form also found in DRG, containing an additional exon [64]. The expression of Na_v_1.7, 1.8 and 1.9 channels is largely restricted to sensory and autonomic peripheral neurons, and Na_v_1.8 and 1.9 are further restricted to small and medium DRG neurons, the majority of which have nociceptive properties.

Alternative splice variants of Na_v_1.7 are generated by the mutually exclusive alternative splicing of exons in human, rat, and mouse. The control of splicing is altered in nerve injury, under which conditions the exon11RS-containing transcripts increase in abundance [65]. Alternative splicing in specific regions of Na_v_1.7 can affect channel properties, such as inactivation and/or reactivation or ramp current properties [66]. In Na_v_1.7 containing a paroxysmal extreme pain disorder mutation (I1461T), multiple properties are altered, including activation, inactivation, closed-state inactivation, and ramp current responses, resulting in increases in numbers of channels available for activation and open probability in these patients [66]. Alternative splice site selection results in the generation of a variant of Na_v_1.8 in DRG neurons 64, 67, with no reported effect of the alternative splicing on channel function [67].

Gain-of function mutations in Na_v_1.7 and 1.9 result in the inherited pain syndromes erythromelalgia [6], paroxysmal extreme pain disorder [66], autosomal-dominant episodic pain [68], and small-fibre neuropathies [6]. Loss-of-function mutations result in congenital insensitivity to pain [69]. Small-fibre neuropathy and congenital insensitivity to pain are also associated with variants of Na_v_1.8 and 1.9, respectively [69]. Some of the known mutations in VGSC in these conditions have been reported to affect splice sites [67]; therefore, alternative splicing is also likely to contribute to such pain syndromes in humans.

### Potassium channels

Potassium channels form the largest ion channel family, and are fundamental to the control of neuronal excitability and the function of multiple cell types. Mutations of potassium channels have profound effects on function, and have been associated with many different conditions and/or diseases, such as epilepsy, ataxia, deafness, hypertension, and hyperinsulinemic hypoglycemia, among many others [70]. There are many different families of potassium channels, including voltage gated, calcium-activated, inwardly rectifying, two-pore, and hyperpolarisation and cyclic nucleotide-activated channels [70]. Although alternatively spliced potassium channels are both predicted and known to exist, considering the diversity of this family, and the important processes in which these channels are involved, it is perhaps surprising that there are many fewer reports of alternative splicing in this family compared with calcium and sodium channels, and none with specific reference to pain and/or nociception 71, 72.

Some of the most likely candidates for study are the two-pore potassium channels, which are expressed in peripheral nociceptive neurons and have been implicated in thermal mechanical and chemical nociception [71]. Splice variants of this family have been described, including N-terminal variants of TREK-1, TREK-2, and TRAAK, and truncated versions of TREK-1 and TRAAK. The truncated TREK-1 and TRAAK channels exert dominant negative effects, form nonfunctional channels, or alter cell surface receptor number; thus, control of splicing in favour of these variants could have therapeutic value 71, 72.

### Transient receptor potential channels

Many transient receptor potential (TRP) channels have been widely studied in relation to somatosensation and pain. The archetypal TRP channel, TRPV1 (a.k.a. the capsaicin receptor) was the first mammalian TRP channel to be cloned, and is intimately related to nociceptive systems, because, in the DRG, it is expressed almost predominantly in nociceptive sensory afferents [73]. It not only transduces noxious heat in sensory afferents, but also interacts with other TRP channels, specifically TRPA1, to sensitise nociceptive afferent terminals to multiple stimuli [74]. Other TRP channels have subsequently been identified and localised to sensory afferents, and are involved in cool and/or noxious cold sensation (TRPM8), warm sensation (TRPV3), mechanical nociception (TRPV4), and very high threshold noxious heat (TRPV2). TRP channels, particularly TRPV1 and TRPA1, are under intensive study as potential drug targets for analgesics [75]. There has been a relatively recent review of the splicing controls of the whole TRP superfamily [76]; thus, here we concentrate only on those channels with particularly well-defined functions of splice variants in nociception: TRPV1 and TRPA1.

Of the four TRPV1 splice variants, three are expressed in DRG or trigeminal ganglia and have some involvement in nociceptive processing [77]. Splicing affects the N-terminal domain in all these splice variants, resulting in loss of activation by capsaicin (in human TRPV1b and TRPV1β[77]) and by other activators, such as protons, resiniferatoxin, and temperature (VR1 5'sv [78]), or can render the channel as a dominant negative (TRPV 5'sv, TRPV1β [79]). These splice variants have functional responses that are different from full-length TRPV1 [79], as might be predicted. When co-expressed with full-length TRPV1, TRPV1 5'sv reduces the TRPV1-current and TRPV1b/β affects TRPV1 function by reduction in channel expression, or destabilisation of heteromeric channels [79].

TRPA1 channels are largely co-expressed, and can interact, with TRPV1 channels in nociceptors [74]. TRPA1 channels have been referred to as universal sensitiser molecules, because they act to sensitise nociceptors to multiple stimuli. One TRPA1 splice variant has been described; this channel alters the cell surface expression of full-length TRPA1 and, when co-expressed with the full-length channel, increases responses to ligand activation. The transcript for this splice variant is regulated in chronic pain states, such as inflammatory and neuropathic pain, suggesting that it contributes to altered neuronal function in these conditions [80].

### Ionotropic glutamate receptors

There are eight splice variants of the NR1 subunit of the NMDA receptor [81]. Inclusion of exons 5 and 21 within the NR1 subunit of the receptor is repressed by a depolarisation-induced Ca^2+^/calmodulin protein kinase (CaMK) pathway, by activation of a sequence known as a CaMK IV-responsive RNA element [11]. Inclusion of exon 5 (NR1-1b variant) reduces the affinity of NR1 for both glutamate and NMDA and potentiates receptor function [81]. Inclusion of exon 21 (NR1-3b) confers calmodulin sensitivity on the resultant channel, binding of which inhibits channel activity [82]. As a result, all of these variants could have significantly different functions compared with other isoforms.

Splice variants including exons 5, 21, and 22 (NR1-1b, NR1-3b and NR1-4b variants respectively) are expressed in the dorsal horn of the spinal cord [83], as is the NR1-4a variant, which is colocalised with NK1 receptors [84]. NK1-expressing neurons in the dorsal horn are nociceptive projection neurons, suggesting that these splice variants are associated with nociceptive processing. Although splicing to NR1-3b is suppressed [11] by neuronal depolarisation, enhanced nociception induced with formalin was not associated with changed expression of any NR1 splice variant [85].

### Acid-sensing ion channels

There are four acid-sensing ion channels (ASIC): ASIC1, ASIC2 (previously MDEG), ASIC3 (previously DRASIC) and ASIC4 (previously SPASIC), some of which have splice variants (ASIC1a/b, ASIC2a/b). ASICs are found in the central (ASIC1a and ASIC2a and b) and peripheral (ASIC1a and b, 2a and b, 3 and 4 [86]) nervous systems in rodents [87]. ASIC channels are fundamentally important in inflammatory pain in both humans and rodents, because they are expressed in somatic and visceral nociceptors, and are activated by low pH [87]. ASIC channels are also expressed in many muscle afferents, implying that they detect metabolic changes, for example, resulting from exercise, and signal pain on tissue acidosis [87]. Given the expression over a large diameter, presumed low threshold sensory afferents, and in nerve endings innervating hair follicles, ASICs have also been hypothesised to have a mechanosensitive function, although this is still under debate [87]. The exact function(s) of ASICs in the CNS are not yet known, but they are also expressed in nociceptive pathways and contribute to opioid analgesia [87].

ASIC1a and ASIC1b differ functionally in that ASIC1b is impermeable to calcium ions [88]; other channel properties are similar between the two splice variants [89]. ASIC2a and 2b differ in that there is an additional transmembrane domain in the N-terminal region of ASIC2b. ASIC2b, which is not activated by protons [90], can form heteromers with both ASIC2a and ASIC3, resulting in channels with more slowly inactivating properties, higher peak currents (with ASIC2a), and channel nonselectivity in the late prolonged phase (with ASIC2a and ASIC3). These sustained currents resulting from heteromers of splice variants might functionally determine nociceptive neuronal responses to sustained low pH, such as in inflammatory conditions.

### P2X receptors

P2X receptors are a family of seven ATP-gated ion channels (P2X1–P2X7), formed from three subunits. In humans, the P2X5 is nonfunctional as a result of a splicing event that removes exon 10; if the exon is replaced, or in humans with a polymorphism in which the necessary intronic splicing site is maintained, the channel regains function [91]. Described P2X variants can have alterations in different regions, some of which are involved in interaction with other proteins, including other P2X subunits, and control of ion permeability [92]. Therefore, P2X splice variants would be predicted to have different functional properties, such as differences in desensitisation [93] or affinity for ATP [94], although others show no obvious difference in functional or pharmacological properties.

The function of many of the P2X receptors has been inferred by expression analysis and phenotypic analysis of mouse KO lines. From these studies, the P2X receptors most strongly implicated in nociception are P2X2, P2X3, P2X4, and P2X7. There is as yet little published evidence of a role for specific splice variants of these four subunits in nociception, other than those of the P2X7 receptor. This is perhaps surprising given the importance of P2X2/3 receptors for the function of primary afferent nociceptors [95].

The role for the P2X7 in nociception, particularly in mechanisms of central sensitisation, is widely recognised, in part as a result of the use of KO mice that showed deficiencies in neuropathic and inflammatory pain behaviours [96]. A recent study on bone cancer pain showed that P2X7 KO mice exhibited more severe pain behaviours than wild-type mice, and expressed the P2X7(k) splice variant in the spinal cord [97]. The P2X7 antagonist A-438079 had no effect on pain behaviours in these animals [97]. The P2X7(k) splice variant has a higher sensitivity to ATP than the ‘classical’ P2X7(a) receptor, and shows sensitivity to ADP-ribosylation not seen in the P2X7(a) variant. The authors concluded that the presence of the P2X7(k) splice variant was unlikely to result in maintained and/or enhanced pain in these animals, because of the use of the same animals to define the important function of P2X7 in chronic pain [97]. Given the different functional properties of the splice variant, it is difficult not to speculate that the P2X7(k) variant could have an, as yet, undefined role in spinal nociceptive processing or in determining pharmacological responses.

### GABA_A_ receptors

The regulation of GABA_A_ receptors contributes to altered nociceptive processing in many experimental pain states [98]. GABA_A_ receptors are highly complex multimeric channels, containing up to 14 subunit types known to contribute to these receptors. The most common variant is the α1β2γ2 type [99]; the gamma subunit confers benzodiazepine responses on GABA_A_ receptors.

Although splice variants of the GABA_A_ beta 4 and gamma 2 subunits were described many years ago 100, 101, nothing has been subsequently published on their possible differential contributions to nociceptive processing. This is surprising considering the importance of GABA_A_ receptors to presynaptic inhibition. GABA_A_ subunit splice variants might confer different properties on the resultant GABA_A_ receptors, for example the short form of the gamma subunit γ2S lacks an additional protein kinase C (PKC) site found in the long form γ2L [100]. In sensory neurons, GABA_A_ channel activity can be strongly modulated by PKC and, hence, presynaptic inhibition can be enhanced or reduced. Therefore, the presence of γ2L in primary afferent nociceptors or spinal neurons could have significant effects on spinal nociceptive processing.

## Growth factors

### Vascular endothelial growth factor

**Figure 3:**
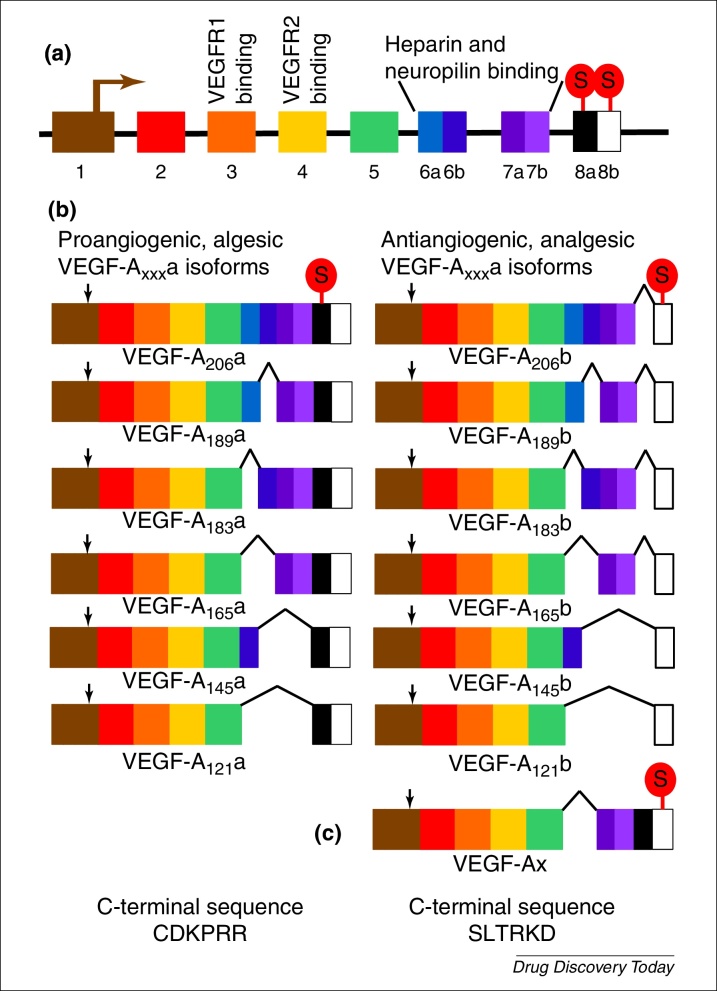
Alternative splicing of the vascular endothelial growth factor A (*VEGFA*) gene. **(a)** Structure of the *VEGFA* gene, showing translational start (denoted by arrow in exon 1) and stop codons, VEGF receptor binding sites, and heparin and neuropilin binding sites. **(b)** Splice variant families of the VEGF-A gene: VEGF_xxx_a and VEGF_xxx_b. The site of the stop codon is shown only in VEGF-A_206_a and b but is in the corresponding location for the other members of each splice variant family. The angiogenic, algesic isoforms of VEGF-A_xxx_a are shown on the left, and the VEGF-A_xxx_b antiangiogenic, analgesic isoforms on the right. Selection of the proximal splice site in exon 8 results in the inclusion of exons 8a and 8b, and use of the proximal stop codon in exon 8a, resulting in a family of splice variants with the C terminal sequence CDKPRR. Use of the distal splice site results in the inclusion of only exon 8b, and thereby introduces a frame shift and use of the different stop codon in exon 8b. The family of splice variants that contains exon 8b has the C terminal sequence SLTRKD. Note that each family comprises several members, all of which contain binding sites for both VEGFR1 and VEGFR2, but which have variable heparin and neuropilin binding. For example, VEGF-A_121_a binds neither heparin nor neuropilin, whereas VEGF-A_165_a binds both heparin and neuropilin. VEGF-A_165_b heparin binding is less than for VEGF-A_189_a but greater than for VEGF-A_145_a because of the differences in exon 6 splicing. **(c)** There is one report in the literature of a further splice variant of VEGF-A, VEGF-Ax, which contains exons 8a and 8b but exhibits translational read-through and, thus, uses the 8b stop codon. Therefore, VEGF-Ax has the VEGF-A_xxx_b C terminal sequence and antiangiogenic activity [124].

Vascular endothelial growth factor (VEGF-A) was not widely recognised for its actions on nociceptive systems until relatively recently [102]. This growth factor is extensively alternatively spliced into two families of splice variants [103]: VEGF-A_xxx_a and VEGF-A_xxx_b (Fig. 3), which have actions on multiple cell types, particularly endothelial cells and neurons. However, many studies overlook the presence of the VEGF-A_xxx_b isoforms despite the observation that they predominate in most normal tissues. Early studies on VEGF-A gave conflicting evidence of its actions on nociceptive processing, with reports of both pro- and antinociceptive effects. This is explained by recent work showing that the two families have algesic (VEGF-A_xxx_a) and analgesic actions (VEGF-A_xxx_b) on peripheral 102, 104 and central nociceptive neurons [105].

### Brain-derived neurotrophic factor

**Figure 4:**
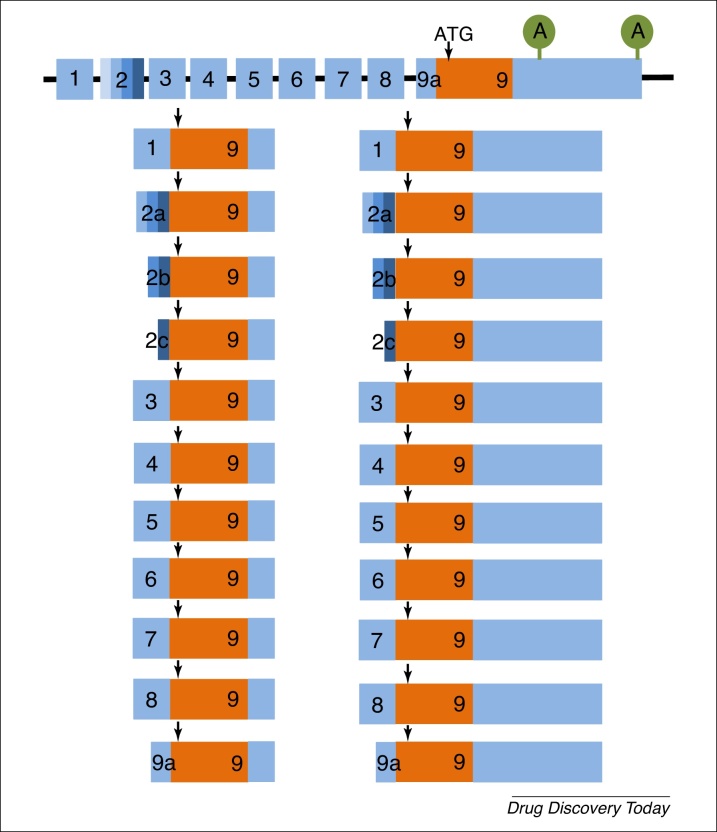
Alternative splicing of the brain-derived neurotrophic factor (BDNF) mRNA. BDNF splicing is unusual in that, although there are at least 22 differently spliced transcripts, all generate the same final peptide. There are at least noncoding 5′ exons (1–8) and a short noncoding sequence in exon 9 (9a) that that can be differentially spliced at the 5-end of the mRNA. In addition, there are three alternative splice sites in exon 2 (denoted by the different shades of blue). The 3-coding exon 9 contains two alternative poly-adenylation sites in exon 9 (denoted by A). The coding sequence (shown in orange) is found in all splice variants. The translation start site (ATG) is denoted by a vertical arrow. At least nine different promoters are present in the *BDNF* gene, and final expression level, regional or subcellular site(s) of expression of BDNF peptide is controlled by differential expression of the different splice variants. Redrawn from [8].

The *BDNF* gene has an unusual splicing pattern in that the 22 described splice variants all generate the same BDNF peptide (Fig. 4). In the case of BDNF, the different splice variants contribute to the control of BDNF expression by differential regulation in different areas of the brain or in other tissues, such as skeletal muscle [8]. In inflammatory pain, only transcripts containing exons 1, 2a, b, c, and 3 increased in primary sensory neurons [106] (Fig. 4). These transcripts, particularly those containing exon 1, are rapidly increased by nerve growth factor (NGF) in culture, suggesting that this potent algesic growth factor exerts effects through control of BDNF differential splicing.

### Nerve growth factor

Both NGF and its receptor TrkA have splice variants 107, 108. The TrkA slice variants do not seem to differ in their functional responses [108] and, surprisingly, there is no published literature relating to different actions of known NGF splice variants in nociception.

## Targeting alternative splicing as a therapeutic strategy

Although alternative splicing has been considered as a potential therapeutic in pathologies such as cancer, eye disease, inherited diseases, such as spinal muscular atrophy, and viral infection 109, 110, 111, 112, to date few clinical trials based on the therapeutic control of alternative splicing have been reported 113, 114. Further trials are reportedly in progress for cholangiocarcinoma (NCT02128282, clinicaltrails.gov), and spinal muscular atrophy (NCT02240355, clinicaltrials.gov). As this review highlights, understanding of alternative splicing and its therapeutic targeting should not be limited to such pathologies, because it can inform or manipulate divergent areas such as physiological processes, pathology, and regional drug differences or adverse effects. Importantly, alternative splicing, as opposed to constitutive splicing, could be an untapped therapeutic approach in areas such as nociception and/or pain, and neuroprotection 102, 105, 115.

Over the past 13 years, several strategies for the control of alternative splicing have been suggested, including targeted oligonucleotides against splice sites [116], or targeting splice factors, such as SR proteins [117].

Chemically modified oligonucleotides resistant to degradation have proved effective at inducing either exon skipping or blocking splice sites in animal models and in some clinical trials, with the aim of repair of defective mRNAs [118]. This approach can result in the restoration of functional protein in conditions such as Duchenne muscular dystrophy, where splicing events result in nonfunctional dystrophin. There has been some minor success in a few clinical trials using this approach. Alternatively, oligonucleotides can be targeted to block aberrant splicing events, restoring functional protein, such as in developing approaches to treat spinal muscular atrophy and beta-thalassemia [118]. Theoretically, these approaches can also be used to control alternative splicing by blocking alternative splice sites to direct splice variant expression; there has been some reported success in this area in animal models [119].

The SR family comprises around 40 proteins that can control multiple steps in RNA processing, including the recognition of splice sites, the binding of RNA polymerase II, mRNA export to the cytoplasm, and translation control [120]. Targeting of CDC-like kinases (Clks) and serine-arginine protein kinases (SRPKs) with small-molecule inhibitors is emerging as a potential therapeutic strategy because these molecules might control limited downstream splicing events, reducing the possibility of off-target or other adverse effects [121]. Proof-of-principle studies have shown efficacy in either blocking exon skipping and enhancing read-through of full-length protein [111], or controlling alternate exon inclusion [122], showing that these strategies might be of use in control of, for example, congenital gain, or loss of function of sodium channels implicated in pain states. Unfortunately, our current understanding of the specific molecules controlling the alternative splicing of specific mRNAs, such as sodium channels, is limited.

The power of controlling alternative splicing is obvious. Splicing directed towards truncated receptors that act as dominant-negative isoforms could be an alternative to antagonists and/or blockers that could reduce adverse effects. Favouring the expression of specific opioid receptor variants could potentiate the analgesic response to an agonist. If directed to ion channels that enhance opioid analgesia, it could improve efficacy of existing drugs. Where families of isoforms exist that have opposite functions, such as VEGF-A, control of splicing between families could shift the balance of isoforms to affect, for example angiogenesis in solid tumours or pain.

To target alternative splicing therapeutically, without affecting constitutive splicing, we need a greater understanding of the specific mechanisms through which alternative splice site selection is controlled for key pre-mRNAs. There have been significant advances in this area, driven largely by the cancer field, but it is an area ripe for development for novel applications, such as the control of pain.

## Conflict of interest

LFD is a co-inventor on patents protecting alternative RNA splicing control and VEGF-A splice variants for therapeutic application in a number of different conditions. LFD is a founder equity holder in, and consultant to, Exonate Ltd, a University of Nottingham spin-out company with a focus on development of alternative RNA splicing control for therapeutic application in a number of different conditions, including analgesia and neuroprotection (www.exonate.com). NB-L has no conflicts of interest. The University of Nottingham also holds equity in Exonate Ltd.
